# The COVID-19 pandemic. Resilience and new needs?

**DOI:** 10.5935/1518-0557.20210014

**Published:** 2021

**Authors:** Maria do Carmo Borges de Souza, Roberto de Azevedo Antunes, Ana Cristina Allemand Mancebo, Marcia Christina Goncalves Gusmao

**Affiliations:** 1 Fertipraxis Centro de Reprodução Humana. Barra da Tijuca, Rio de Janeiro, RJ, Brazil

That’s it. We face a mixture of facts, reasons and feelings, science and emotion. Each day brings us reflection and decision-making. Today’s data shows 2,589,638 global deaths, 681,703 among our REDLARA’s countries (John Hopkins University, 2021). These are painful losses, each one unique on its own, and all of them dearly felt by us all. We are undoubtedly experiencing challenges never before imagined. Even though the light in the end of the tunnel draws near with the arrival of the vaccines, difficulties related to its manufacturing and distribution, as well as the appearance of viral variants, make the end of this pandemic still far away. So, being an IVF staff demands, more than anything, mental and physical strength, resilience and care to our patients and their families.

Historically the “Black Death” plague was responsible for widespread pandemic with high mortality during the fourteenth century, causing more than 50 million deaths throughout Europe (WHO, 2017). Today, knowledge has enabled better ways to fight the SARS CoV-2, but the challenges persist, from facing the pandemic itself as well as providing information to the lay public, demystifying concepts for some who act as they were back to the times of the plague and obscurantism.

At the same time, how to continue living in this moment without a horizon of normalcy, how to seek safe expectations? How to welcome our patients who wish to begin their families? We can quote Churchill, who said, “even if we are going through hell, we shall keep on going”. Our resilience is being tested, we resist, or to keep on with the prime minister’s words: “We shall never surrender”. Initiatives from world reproductive societies, such as REDLARA and SBRA, have enabled the spread of knowledge and a broad strengthening of scientific ties among peers.

In the early stages of the COVID-19 pandemic, IVF clinics stopped patient treatment cycles to minimize the risk of disease transmission. Infertility does not wait, and during the pandemic, there has been a sharp increase in the prevalence of stress and depression among infertile patients (Ben-Kimhy *et al*., 2020). As soon as possible, prevention through the preservation of oocytes grew as an important strategy, while the use of frozen oocytes increased significantly. Embryo transfers resumed worldwide. Furthermore, the risk of SARS-CoV-2 viral exposure and potential cross contamination within the IVF lab has been addressed by many studies, demonstrating the possibility of safe laboratory operations, including cryostorage (Huang *et al*., 2021). The COVID-19 pandemic forced a new way of working in face of the new challenges. All teams must work in constant care, acting properly to avoid virus dissemination. Additional stress arises from the fact that often teammates and their families develop COVID-19 and this situation requires making decisions that at the same time include necessary care and support for the team and maintenance of careful work environment both in the clinic and in the laboratory (Rajput *et al*., 2021).

At this moment, we do not have a clear understanding about the length and implications of the pandemic. Meanwhile we experience different waves of dissemination, it is clear that treatments should consider specific local conditions and that our patients will not give up easily.

It is also time to focus on prevention and psychological care, not only for patients but also for all team members exposed to distress, tiredness and burnout. Although we are living in a globally connected world where lots of support applications and Internet technologies appear all the time, it seems that the “new needs” for the healthcare team and patients are nothing more than the well-known “old needs” - to listen and care!
